# HIF‐1α/VEGF signaling‐mediated epithelial–mesenchymal transition and angiogenesis is critically involved in anti‐metastasis effect of luteolin in melanoma cells

**DOI:** 10.1002/ptr.6273

**Published:** 2019-01-17

**Authors:** Chunyu Li, Qi Wang, Shen Shen, Xiaolu Wei, Guoxia Li

**Affiliations:** ^1^ Department of Integrated Chinese Traditional and Western Medicine, International Medical School Tianjin Medical University Tianjin China; ^2^ Department of Oncology Shanghai Pulmonary Hospital Affiliated Tongji University Shanghai China

**Keywords:** angiogenesis, epithelial‐mesenchymal transition, HIF‐1α/VEGF signaling, luteolin, melanoma, metastasis

## Abstract

Tumor metastasis is still the leading cause of melanoma mortality. Luteolin, a natural flavonoid, is found in fruits, vegetables, and medicinal herbs. The pharmacological action and mechanism of luteolin on the metastasis of melanoma remain elusive. In this study, we investigated the effect of luteolin on A375 and B16‐F10 cell viability, migration, invasion, adhesion, and tube formation of human umbilical vein endothelial cells. Epithelial–mesenchymal transition (EMT) markers and pivotal molecules in HIF‐1α/VEGF signaling expression were analysed using western blot assays or quantitative real‐time polymerase chain reaction. Results showed that luteolin inhibits cellular proliferation in A375 and B16‐F10 melanoma cells in a time‐dependent and concentration‐dependent manner. Luteolin significantly inhibited the migratory, invasive, adhesive, and tube‐forming potential of highly metastatic A375 and B16‐F10 melanoma cells or human umbilical vein endothelial cells at sub‐IC_50_ concentrations, where no significant cytotoxicity was observed. Luteolin effectively suppressed EMT by increased E‐cadherin and decreased N‐cadherin and vimentin expression both in mRNA and protein levels. Further, luteolin exerted its anti‐metastasis activity through decreasing the p‐Akt, HIF‐1α, VEGF‐A, p‐VEGFR‐2, MMP‐2, and MMP‐9 proteins expression. Overall, our findings first time suggests that HIF‐1α/VEGF signaling‐mediated EMT and angiogenesis is critically involved in anti‐metastasis effect of luteolin as a potential therapeutic candidate for melanoma.

## 1 INTRODUCTION

Melanoma originates in the pigment‐producing melanocytes and is the most lethal skin‐related cancer (Turner, Ware, & Bosenberg, 2018). It is the one of the tumors that most frequently disseminate through metastasis spread to distant site. Despite advances in the detection and treatment of melanoma, metastasis remains the leading cause of the skin‐related death (Zivadinovic et al., 2016). Prevention of cancer metastasis at early stages is a key factor for improving patient survival (Becker et al., 2017). Therefore, the exploration of therapeutic candidate for melanoma metastasis is very attractive to cancer research.

Melanoma progression toward invasive and metastatic is associated with the reactivation of epithelial‐mesenchymal transition (EMT), which involves epithelial traits loss and mesenchymal characteristics acquisition (Yi et al., 2018). A typical phenotype of EMT is usually associated with downregulation of E‐cadherin, upregulation of N‐cadherin and vimentin, resulting in weakened adhesion ability and enhanced motility (Ryu et al., 2017). In addition, tumor angiogenesis, the growth of new blood vessels, also plays a key role in the development, growth, and migration of melanoma cells and may also facilitate pathways for dissemination during the process of metastasis (Wang et al., 2015). Vascular endothelial growth factor (VEGF) and related VEGF receptors have a central role in the modulation of pathological angiogenesis (Ni et al., 2012). Furthermore, VEGF binds to its receptor activating VEGFR2, which further leads to the secretion of matrix metalloproteinases (MMPs), resulting in the degradation of extracellular matrix (ECM) and provision of passage for cells to invade the nearby tissue. MMP‐2 and MMP‐9 are closely related to the migratory and invasive ability of cancer cells and present in various malignant tumors (Botti et al., 2013). In addition, VEGF is the key downstream effector of hypoxia‐inducible factor‐1α (HIF‐1α) and plays key roles in inducing cell migration, proliferation, and tube formation with a unique specificity for endothelial cells. Moreover, HIF‐1α was shown to induce EMT in many types of cancer tissues. HIF‐1α expression profile was correlated with the expression levels of E‐cadherin, N‐cadherin, and vimentin (Lai et al., 2016). Based on these findings, HIF‐1α/VEGF signal pathway is considered to be an important target for the treatment of angiogenesis‐related diseases including metastasis of cancer.

Luteolin (3′, 4′, 5, 7‐terahydroxyflavone) is a common effective dietary flavonoid that is present in fruits, vegetables, and medicinal herbs (Liu, Xu, Yan, Cheng, & Liu, 2018; Song et al., 2017). Furthermore, accumulating evidences have suggested that luteolin possesses multiple pharmacological activities such as anti‐inflammation, anti‐hypertension, antioxidant, and antitumor, which are also supposed to be the basis of these biological properties (Aziz, Kim, & Cho, 2018; Cui et al., 2017). Previous studies indicate that luteolin suppress cancer cell growth through multiple mechanisms including promoting cancer cell apoptosis and cell cycle arrest, inhibiting cell proliferation and tumor growth, improving drug resistance, and mitigating invasiveness and metastasis of cancer cells (Cook, Liang, Besch‐Williford, & Hyder, 2017; Wang et al., 2014). Specifically, it has been found that luteolin inhibited the tumor formation in vivo through its antioxidant and anti‐inflammatory activity (Hwang, Lee, Kim, & Hwang, 2013). Furthermore, luteolin effectively blocked progestin‐dependent angiogenesis and the stem cell‐like phenotype in human breast cancer cells (Cook, 2018). Although these studies revealed luteolin's protective roles in cancer, the effects and underlying mechanisms of luteolin on the metastasis of melanoma have not been reported previously.

Therefore, in the present study, we evaluated the effects of luteolin on A375 and B16‐F10 cell viability, migration, invasion, adhesion, and tube formation of human umbilical vein endothelial cells (HUVECs). Concurrently, the underlying molecular mechanism of luteolin anti‐metastasis were investigated in association with inhibition of HIF‐1α/VEGF signaling pathway and the resulting decreased EMT and angiogenesis.

## 2 MATERIALS AND METHODS

### 2.1 Reagents and cell culture

Luteolin (99.9% purity, CAS No. 491–70‐3) was purchased from Ark Pharm, Inc. (Illinois, USA) and was dissolved in dimethyl sulfoxide (DMSO, Sigma, St Louis, MO, USA) as 100‐mM stock solution (stored at 4°C) for experiments. 3‐(4,5‐dimethyl‐2‐thiazoyl)‐2,5‐diphenyl‐2H‐ tetrazolium bromide (MTT) and LY294002 (PI3K inhibitor) were obtained from Sigma‐Aldrich (St Louis, MO, USA). Dulbecco's modified eagle's medium (DMEM), fetal bovine serum (FBS), and penicillin/streptomycin were purchased from Gibco (Thermo Fisher Scientific Inc, USA). Antibodies against E‐cadherin (mouse; cat. no. 14472), N‐cadherin (rabbit; cat. no. 13116), and vimentin (mouse; cat. no. 3390) were purchased from Cell Signaling Technology (Cambridge, MA, USA). Primary antibodies to Akt (rabbit; cat. no. ab235958), p‐Akt (rabbit; phosphor S473, cat. no. ab81283), HIF‐1α (rabbit; cat. no. ab51608), VEGF‐A (rabbit; cat. no. ab46154), VEGFR‐2 (rabbit; cat. no. ab2349), p‐VEGFR‐2(rabbit; phosphor Y951, cat. no. ab38473), MMP‐2 (rabbit; cat. no. ab37150), MMP‐9 (rabbit; cat. no. ab73734), and β‐actin (rabbit; cat. no. ab8227) were purchased from Abcam (Cambridge, MA, USA). Goat anti‐mouse IgG‐HRP (cat. no. sc‐2005) and goat anti‐rabbit IgG‐HRP (cat. no. sc‐2004) were purchased from Santa Cruz Biotechnology (Dallas, TX, USA).

Human melanoma A375 obtained from American Type Culture Collection (ATCC, Manassas, VA, USA). Mouse melanoma B16‐F10 and HUVECs were purchased from the Cell Culture Center of Chinese Academy of Medical Sciences (Beijing, China). The cells were cultured in DMEM containing 10% FBS, 100‐U/mL penicillin, and 100‐μg/mL streptomycin. Hypoxia was induced by exposing the cells to cobalt chloride (CoCl_2,_ Sigma‐Aldrich, St. Louis, MO, USA). Cells were seeded into 6‐well plates at a density of 1 × 10^5^/well. After 90% confluence was reached, they were treated with CoCl_2_ at concentration 100 μM in DMEM medium with 0.5% FBS for 24 hr (Tatrai et al., 2017). All cell cultures were maintained at 37°C in a humidified atmosphere of 5% CO_2_.

### 2.2 Cell viability assay

Cell viability was determined by MTT assay to investigate the cytotoxic effect of luteolin in vitro (Corina et al., 2017). A375 and B16‐F10 cells were seeded into 96‐well culture plates at the density of 5 × 10^3^ cells per well. When the cells were adherent to the walls, the cells were treated with various concentrations (5, 10, 20, 40, and 60 μM) of luteolin for 24, 48, and 72 h. The cultural medium was removed, and 100 μL of MTT (1 mg/mL) was added to each well. After incubating for 4 hr, the formazan crystals were dissolved in 150‐μL DMSO and then measured the absorbance of each well with a microplate reader (BioTek Epoch, VT, USA) at 490 nm. The test was repeated three times. Inhibition rate (% of control) = (1‐absorbance of test sample/absorbance of control) × 100%.

### 2.3 Wound‐healing assay

Cell migration was measured by wound‐healing assay to evaluate the inhibit migration effect of luteolin in vitro (Si et al., 2018). A375 and B16‐F10 cells were seeded in 60‐mm dishes at the density of 8 × 10^5^ cells per dish to 90–100% confluence. In the center of each dish, a denuded zone was carefully created by scraping the cell monolayer with pipette tip. After wounding with pipette tip, the cells were washed with phosphate buffered saline, and serum‐free medium containing various concentrations (5, 10, and 20 μM) of luteolin had been added. Then, the cells were allowed to migrate for 24 hr. At predetermined time points (0, 3, 6, 9, 12, and 24 hr), the widths of wound were measured, and images of cells that migrated into the wounded region were captured at time 0 and 24 hr using an inverted microscope (IX71, Olympus, Tokyo, Japan).

### 2.4 Transwell invasion assay

Cell invasion was detected using Matrigel‐coated transwell chambers (8 μm pore size; Corning, USA) assay according to the manufacturer's instructions (Si et al., 2018). After pretreatment with or without (control) various concentrations (5, 10, and 20 μM) of luteolin for 24 hr, A375 and B16‐F10 cells were harvested and seeded into the upper chamber at the density of 4 × 10^4^ cells per well in serum free medium. The lower chambers were filled with DMEM medium supplemented with 10% FBS. The cells were allowed to invade for 24 hr at 37°C in a humidified 5% CO_2_ atmosphere. The invading cells were fixed with methanol for 20 min and stained with Giemsa solution for 20 min. Cell numbers in five separate fields were counted and photographed using an inverted microscope with 100× magnification (IX71, Olympus, Tokyo, Japan).

### 2.5 Cell adhesion assay

After pretreatment with or without (control) various concentrations (5, 10, and 20 μM) of luteolin for 24 hr, A375 and B16‐F10 cells were harvested and seeded into the 24‐well plates coated with fibronectin (10 ng/mL) at the density of 2 × 10^5^ cells per well. After further incubations for 5, 15, and 30 min, nonadherent cells were removed by phosphate buffered saline washes. The adherent cells were fixed with methanol and counted in five separate fields under a light microscope with 200× magnification (Olympus, Tokyo, Japan).

### 2.6 Tube formation assay

The HUVECs capillary‐like formation assay was performed on Matrigel (BD Biosciences, MA, USA). Matrigel was added into 96‐well plate and cultured in 37°C for 2 hr to make Matrigel freeze. After pretreatment with or without (control) various concentrations (5, 10, and 20 μM) of luteolin for 24 hr, HUVECs were seeded in plates at the density of 8 × 10^3^ cells per well. After 6 hr incubation, morphological changes of the cells and tube formation were observed under a phase‐contrast microscope and photographed at 200× magnification.

### 2.7 Quantitative real‐time polymerase chain reaction

After pretreatment with or without (control) various concentrations (5, 10, and 20 μM) of luteolin for 24 hr, the total RNA of A375 and B16‐F10 cells was extracted by TRIzol reagent (Life Technologies; Thermo Fisher Scientific, Inc.). Reverse transcription was performed with fast quant RT kit (Tiangen Biotech Co., Ltd., Beijing, China) according to the manufacturer's protocol. Real‐time polymerase chain reaction (PCR) mixture volume was 25 μL, including 12.5‐μL SYBR green mix, 0.2‐μL cDNA, 1.5‐μL primers per mix (10 μM each primer), and 10.8‐μL RNAse‐free H_2_O. The experiment was then set up with the following PCR program on ABI 7500 (Applied Biosystems Inc. USA): 95°C for 15 min, 1 cycle; 40 cycles of 95°C for 10 s, 60°C for 20 s, 72°C for 30 s. Specific primers were designed by gene runner software (version 6.5.48) and were synthesized by Beijing Aoke Biotechnology Co., Ltd (Beijing, China). The specific primers are shown in Table 1. The relative quantification of gene expression was calculated using the 2^‐ΔΔCt^ method and presented as a proportion of targeted gene expression to control gene (β‐actin). All assays were performed in triplicate and independently repeated three times.

**Table 1:** Primers sequences used in quantitative polymerase chain reaction

Gene name	Primer sequence (5′‐3′)
E‐cadherin	F: GGATTGCAAATTCCTGCCATTC
	R: AACGTTGTCCCGGGTGTCA
N‐cadherin	F: GAGAGGAAGACCA‐GGACTATGA
	R: CAGTCATCACCACCACCATAC
Vimentin	F: GCAGGAGGCAGAAGAATGGTA
	R: GGGACTCATTGGTTCCTTTAAGG
β‐actin	F: CCACGAAACTACCTTCAACTCCA
	R: GTGA TCTCCTTCTGCATCCTGTC
F, forwar; R, reverse	

### 2.8 Western blotting assay

Western blotting assay was used for detection of E‐cadherin, N‐cadherin, vimentin, Akt, p‐Akt, HIF‐1α, VEGF‐A, VEGFR‐2, p‐VEGFR‐2, MMP‐2, and MMP‐9 expression levels in A375 and B16‐F10 cells. Following pretreatment with or without (control) various concentrations (5, 10, and 20 μM) of luteolin for 24 hr, the protein lysates from cultured cells were separated by 10% SDS‐PAGE systems and transferred to polyvinyllidene difluoride membranes (Millipore, USA). After blocking with 5% skim milk in tris‐buffered saline containing 0.1% Tween 20 for 2 hr, the membranes were incubated with primary antibodies at 1:500–1:1,000 dilutions with 5% BSA in TBST overnight at 4°C. The antibodies and dilution factors were as follows: E‐cadherin (dilution, 1:1,000), N‐cadherin (dilution, 1:1,000), vimentin (dilution, 1:1,000), Akt (dilution, 1:1,000), p‐Akt (dilution, 1:1,000), HIF‐1α (dilution, 1:500), VEGF‐A (dilution, 1:800), VEGFR‐2 (dilution, 1:800), p‐VEGFR‐2 (dilution, 1:800), MMP‐2 (dilution, 1:800), MMP‐9 (dilution, 1:800), and β‐actin (dilution, 1:600). The blots were washed and incubated with secondary antibodies conjugated with horseradish peroxidase (HRP) and incubated for 1 hr at room temperature. Membranes were visualized using enhanced chemiluminescence (Millipore, USA) and were photographed using G‐BOX (Gene Company Ltd, Beijing, China). Bands analyzed by Image Pro Plus 6.0 software (Media Cybernetics Inc., MD, USA).

### 2.9 Statistical analyses

Statistical analysis was performed using SPSS 19.0 (Chicago, IL, USA). All data are presented as the mean ± standard deviation (SD). One‐way analysis of variance test was utilized to analyze the difference between the groups. The least significant difference and Tukey method were utilized to analyze post hoc multiple comparisons. *P* values were two‐sided; *p* < 0.05 was considered to indicate a statistically significant difference.

## 3 RESULTS

### 3.1 Luteolin‐inhibited viability of A375 and B16‐F10 cells

**Figure 1:**
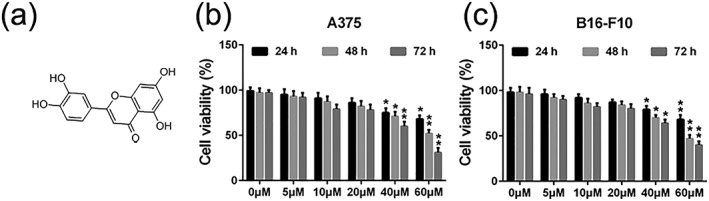
Effects of luteolin on the viability of melanoma cells A375 and B16‐F10. (a) Chemical structure of luteolin. (b) A375 and (c) B16‐F10 cells were treated with indicated concentrations of luteolin (5, 10, 20, 40, and 60 μM) for 24, 48, and 72 hr, then cell viability was measured by MTT assay. Values are the means ± SD from three independent determinations. *p < 0.05 and **p < 0.01 indicate a significant difference from the control group

Luteolin has a molecular weight of 286.24 g/mol, and its molecular structure is shown in Figure 1a. Firstly, we measured the inhibitory effects of luteolin on the viability of melanoma cells A375 and B16‐F10 by MTT assay. Figure 1b,c showed that luteolin decreased cell viability in a time‐dependent and concentration‐dependent manner in both A375 and B16‐F10 cells. IC_50_ values of luteolin after 24, 48, and 78 hr of incubation were 140.73, 64.94, and 44.45 μM for A375 cells and 143.89, 67.34, and 55.09 μM for B16‐F10 cells, respectively. Luteolin inhibited the growth of A375 and B16‐F10 cells in the presence of 40 and 60 μM after 24, 48, and 72 hr incubation, whereas 0–20 μM of luteolin did not significantly decrease cellular viability after 24, 48, and 72 hr of incubation respectively. Thus, in the subsequent experiments, we chose the concentration 5, 10, and 20 μM of luteolin for further investigation in order to exclude the luteolin's cytotoxicity affect cell's metastatic ability.

### 3.2 Luteolin inhibited the migratory potential of A375 and B16‐F10 cells

**Figure 2:**
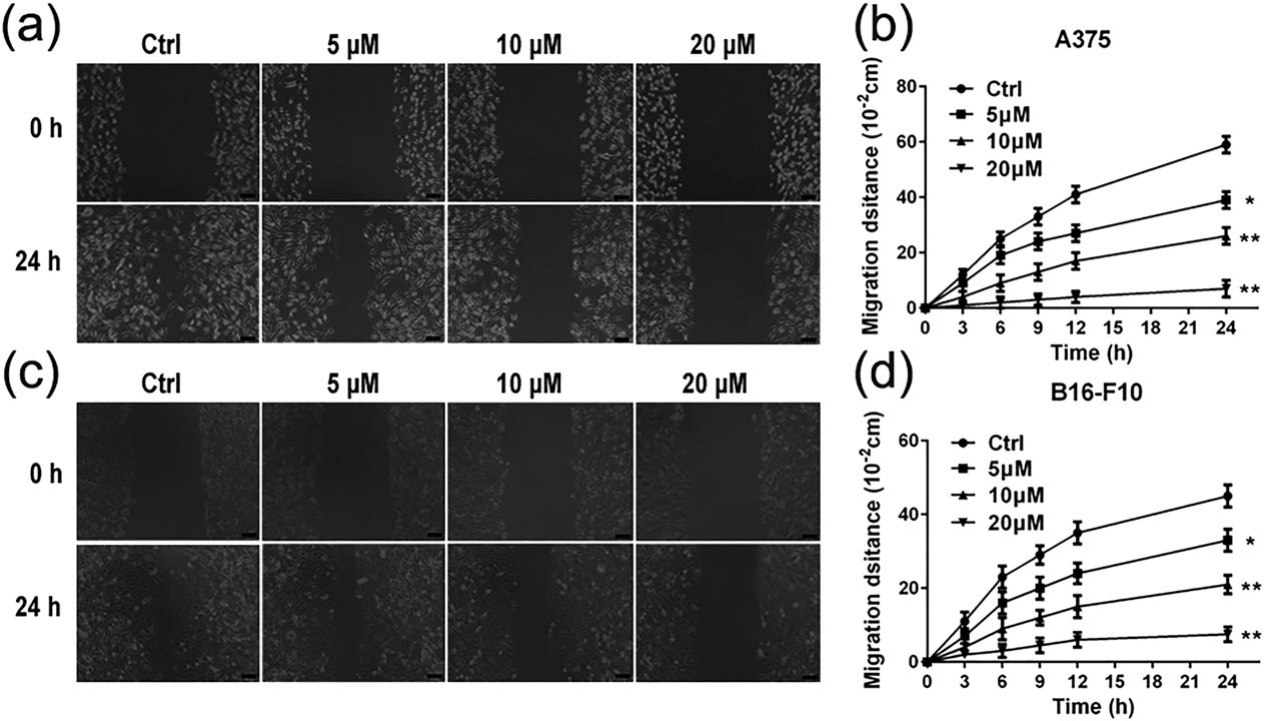
Effects of luteolin on the migratory ability of melanoma cells. (a and b) A375 and (c and d) B16‐F10 cells were treated with indicated concentrations (5, 10, and 20 μM) of luteolin for 24 hr. Then, the inhibitory effects of luteolin on A375 and B16‐F10 cell migration was evaluated by wound‐healing assay. Images were taken at 0 and 24 hr after the wound scratch area was made under the invert microscope (100× magnification). The scale in the figure is 100 μm. The migration distance was measured by the scale of microscope. Values are the means ± SD from three independent determinations. *p < 0.05 and **p < 0.01 indicate a significant difference from the control group

Cell migration is essential step in cancer metastasis. As shown in Figure 2a–d, in the absence of luteolin (control group), A375 and B16‐F10 cells displayed high migrated capabilities as indicated by being able to completely heal the wound scratch. The activity of migration of A375 and B16‐F10 cells was markedly suppressed by luteolin in a concentration‐dependent manner.

### 3.3 Luteolin inhibited the invasion of A375 and B16‐F10 cells

**Figure 3:**
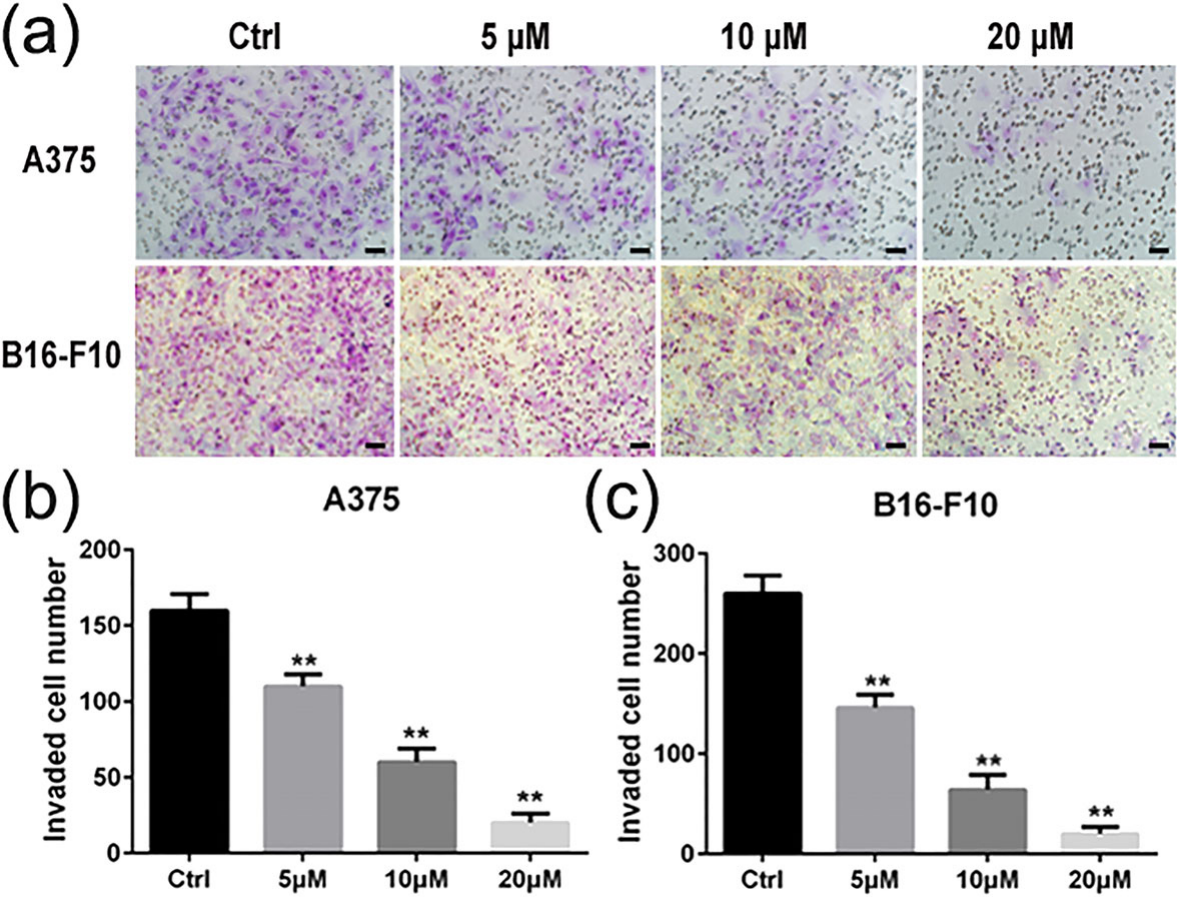
Effects of luteolin on the invasion ability of melanoma cells. A375 and B16‐F10 cells were treated with indicated concentrations of luteolin (5, 10, and 20 μM) for 24 hr, and the number of invasive cells was determined using a transwell matrix penetration assay. (a) A375 and B16‐F10 cells, which invaded were fixed with methanol and stained with Giemsa. Cell numbers were counted in five separated fields. Cell numbers in five fields were counted for each slide under the microsope with 200× magnitudes. The scale in the figure is 100 μm. (b and c) values are the means ± SD from three independent determinations. *p < 0.05 and **p < 0.01 indicate a significant difference from the control group [Colour figure can be viewed at wileyonlinelibrary.com]

Cell invasion was measured by matrigel‐coated transwell chambers assay to investigate the inhibitory effect of luteolin on the invasion of A375 and B16‐F10 cells. According to the results in Figure 3a–c, A375 and B16‐F10 cells displayed high invasive potential as indicated by being able to completely penetrate through the matrigel‐coated filters in the absence of luteolin (control group). The activity of invasion of A375 and B16‐F10 cells was markedly suppressed by luteolin in a concentration‐dependent manner.

### 3.4 Luteolin inhibited A375 and B16‐F10 cells adhesion

**Figure 4:**
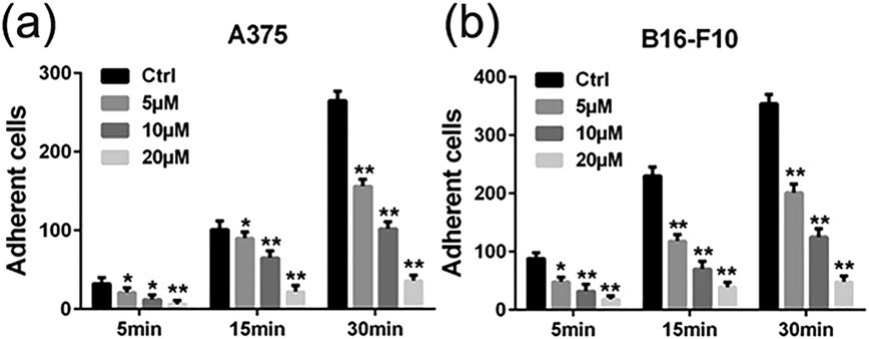
Effects of luteolin on the adhesion ability of melanoma cells. (a) A375 and (b) B16‐F10 cells were pretreated with indicated concentrations (5, 10, and 20 μM) of luteolin for 24 hr, followed by measuring adhesion capacity on fibronectin over indicated time periods. Cell numbers in five fields were counted for each slide under the microsope with 200× magnitudes. Values are the means ± SD from three independent determinations. *p < 0.05 and **p < 0.01 indicate a significant difference from the control group

Adhesion of cancer cells to components of the ECM is an essential step for cancer metastasis. Therefore, we measured the effect of luteolin on cell adhesion. In the absence of luteolin (control group), A375 and B16‐F10 cells displayed high adhesive capabilities as indicated by being able to completely adhere to the fibronectin (Figure 4a,b). These data revealed that luteolin markedly suppresses A375 and B16‐F10 cells activity of adhesion in a concentration‐dependent manner (Figure 4a,b).

### 3.5 Luteolin inhibited the tube formations of HUVECs cells in vitro

**Figure 5:**
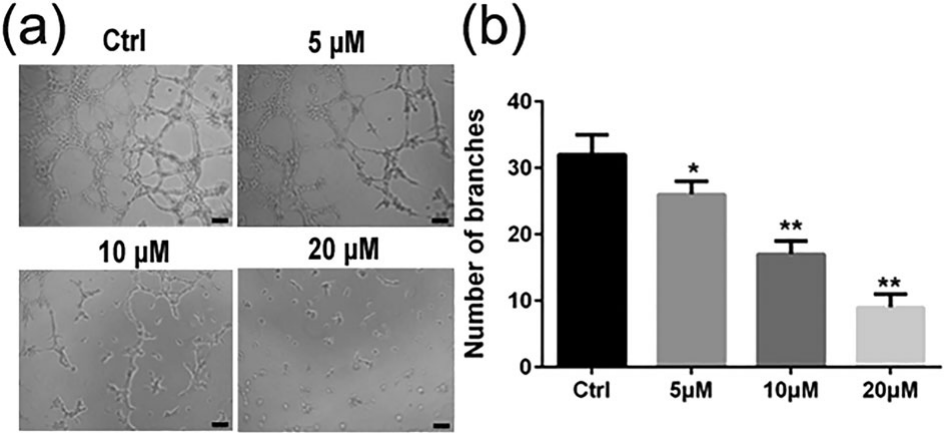
Effects of luteolin on the capillary tube formation of HUVECs. (a and b) HUVECs were pretreated with indicated concentrations (5, 10, and 20 μM) of luteolin for 24 hr. Capillary tube formation was assessed after 6 hr. Images were taken, and the total number of nodes and branches were calculated under a phase‐contrast microscope with 200× magnitudes. The scale in the figure is 100 μm. Values are the means ± SD from three independent determinations. *p < 0.05 and **p < 0.01 indicate a significant difference from the control group

Angiogenesis, the key step in tumor growth and metastasis, provides the necessary oxygen and nutrients for tumor and may also facilitate pathways for dissemination during the process of metastasis. To investigate the effect of luteolin on angiogenesis in vitro, the capillary tube formation of HUVECs cells on Matrigel was performed. As shown in Figure 5a,b, the capillary tube formation of HUVECs cells was markedly inhibited after 24 hr exposure to luteolin when compared with the control group. These results indicated that luteolin had a concentration‐dependent inhibitory effect on the tube formation of HUVECs cells in vitro.

### 3.6 Luteolin suppressed protein and gene expression of EMT typical markers in A375 and B16‐F10 cells

**Figure 6:**
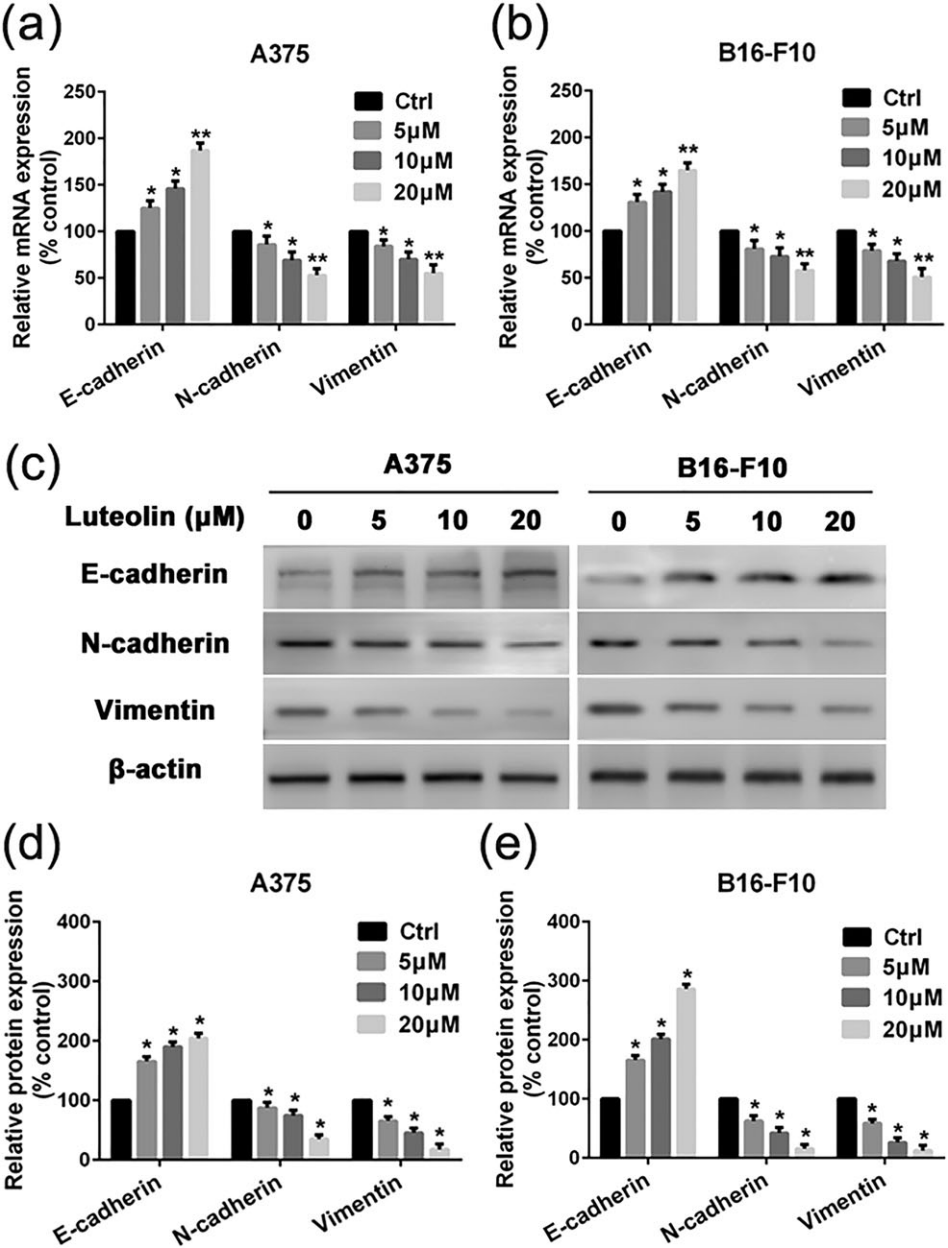
Effects of luteolin on EMT‐related marker's mRNA and protein expression. (a) A375 and (b) B16‐F10 cells were pretreated with indicated concentrations of luteolin (5, 10, and 20 μM) for 24 hr, followed by quantitative real‐time polymerase chain reaction assay to measure the regulatory effect of luteolin on mRNA expression of E‐cadherin, N‐cadherin, and Vimentin. (c) A375 and B16‐F10 cells were pretreated with indicated concentrations of luteolin for 24 hr, followed by western blotting assay to test the protein expression of E‐cadherin, N‐cadherin, and Vimentin. β‐actin was used as loading control. (d and e) Relative protein expression for all proteins qualified using Image Pro Plus software, respectively. Values are the means ± SD from three independent determinations. *p < 0.05 indicate a significant difference from the control group

Accumulating evidence indicates that metastasis is a programmed multistep process. EMT is one purported mechanism linked to tumor invasion and metastasis, which is characterized by loss of polarity, reduced cell–cell adhesion, and increased cell motility. To further confirm the effects of luteolin on EMT, we measured the expression of three EMT typical markers including E‐cadherin, N‐cadherin, and vimentin by qRT‐PCR and western blotting. The data demonstrated that the effects of luteolin are concentration‐dependent increasing the expression of E‐cadherin and decreasing the expression of N‐cadherin and vimentin in A375 and B16‐F10 cells compared with the control group both in protein and gene levels (Figure 6a–d).

### 3.7 Luteolin inhibited the HIF‐1α/VEGF signaling pathway in A375 and B16‐F10 cells

**Figure 7:**
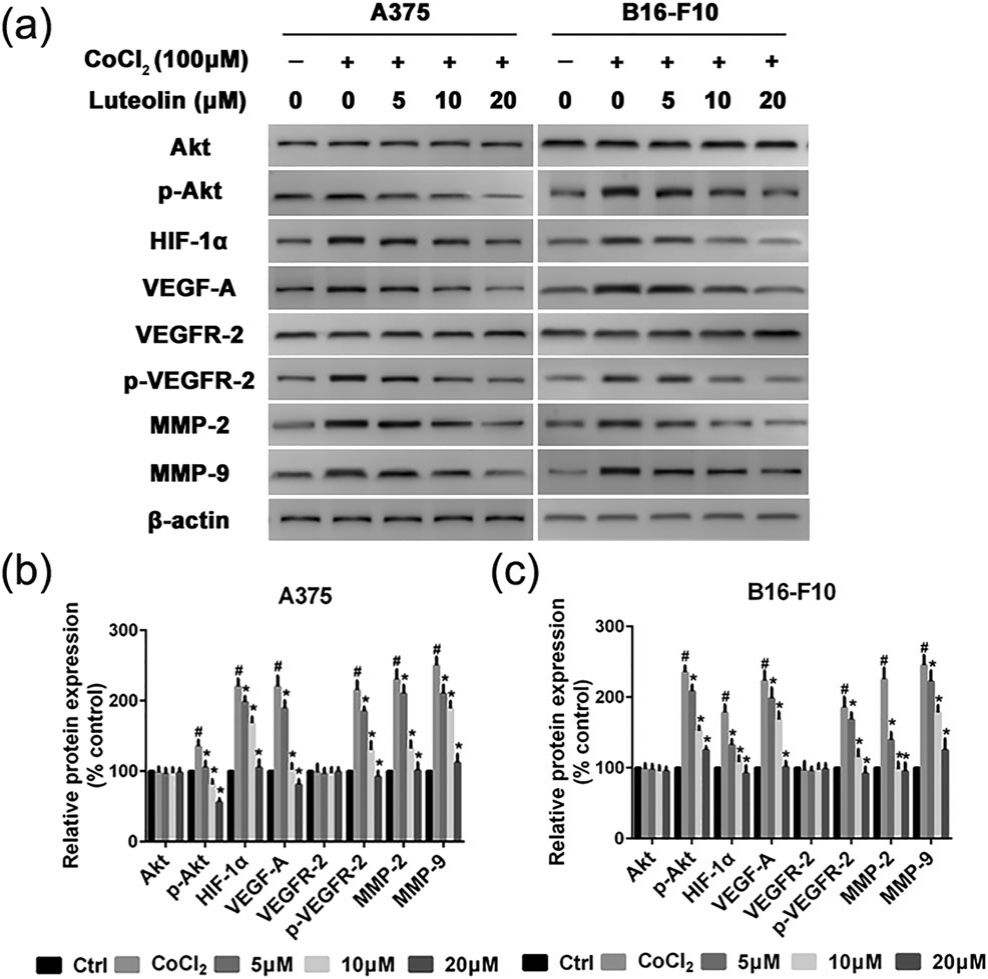
Effects of luteolin on the protein's expression of HIF‐1α/VEGF pathway. (a) A375 and B16‐F10 cells were pretreated with indicated concentrations (5, 10, and 20 μM) of luteolin for 24 hr, followed by western blotting assay to test the protein expression of Akt, p‐Akt, HIF‐1α, VEGF‐A, VEGFR‐2, p‐VEGFR‐2, MMP‐2, and MMP‐9. β‐actin was used as loading control. (b and c) Relative protein expression for all proteins qualified using Image Pro Plus software, respectively. Values are the means ± SD from three independent determinations. *p < 0.05 indicate a significant difference from the hypoxia (CoCl_2_) group. ^#^ *p* < 0.05 indicate a significant difference from the control group

**Figure 8:**
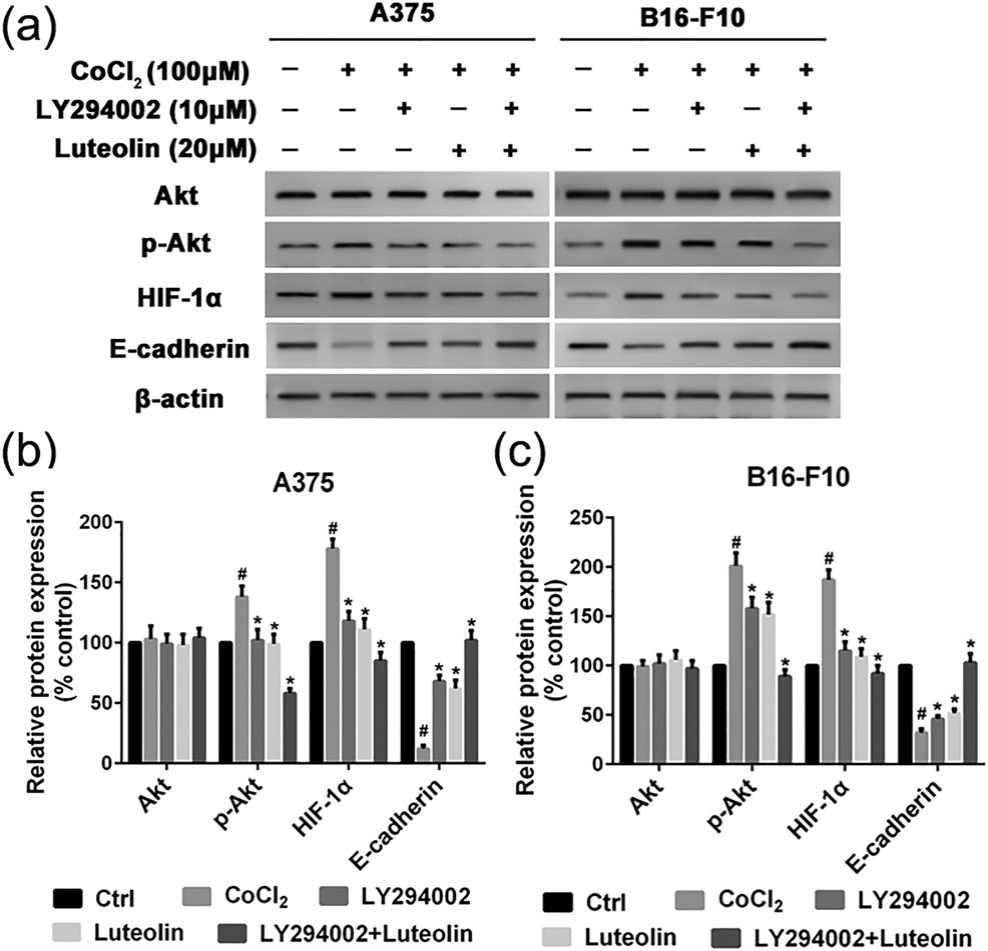
Luteolin and LY294002 inhibit EMT via the Akt/HIF‐1α pathway. (a) A375 and B16‐F10 cells were pretreated with indicated concentrations of luteolin and LY294002 for 24 hr, followed by western blotting assay to test the protein expression of Akt, p‐Akt, HIF‐1α, and E‐cadherin. β‐actin was used as loading control. (b and c) Relative protein expression for all proteins qualified using Image Pro Plus software, respectively. Values are the means ± SD from three independent determinations. *p < 0.05 indicate a significant difference from the hypoxia (CoCl_2_) group. ^#^ *p* < 0.05 indicate a significant difference from the control group

HIF‐1α/VEGF signal pathway is considered to be an important target for inhibition of EMT and antiangiogenic therapies to treat cancer disease. Thus, we detected the expressions of key molecules in HIF‐1α/VEGF signal pathway, including Akt, p‐Akt, HIF‐1α, VEGF‐A, VEGFR‐2, p‐VEGFR‐2, MMP‐2, and MMP‐9 in A375 and B16‐F10 cells by western blotting. CoCl_2_ is a chemical hypoxia agent that leads to the stabilization of HIF‐1α and the expression of hypoxia responsive genes. Indeed, we observed the upregulation of HIF‐1α in A375 and B16‐F10 cells after incubation with CoCl_2_ at concentration of 100 μM for 24 hr. The protein expression level of HIF‐1α was rapidly upregulated. The data also showed that the levels of p‐Akt, VEGF‐A, p‐VEGFR‐2, MMP‐2, and MMP‐9 were significantly upregulated by exposure to CoCl_2_ (Figure 7a,b). Luteolin could evidently suppress the expression of p‐Akt, HIF‐1α, VEGF‐A, p‐VEGFR‐2, MMP‐2, and MMP‐9, whereas the total expressions of Akt and VEGFR‐2 were almost unaffected (Figure 7a,b). These results indicated that luteolin may inhibit HIF‐1α/VEGF associated signal pathway and the resulting decreased EMT and angiogenesis (Figure 7a,b). Next, in order to verify luteolin suppress metastasis due to Akt/HIF‐1α/EMT signaling pathway, A375 and B16‐F10 cells were pretreated with indicated concentrations of luteolin and LY294002 for 24 hr, followed by western blotting assay to test the protein expression of Akt, p‐Akt, HIF‐1α, and E‐cadherin. As shown in Figure 8, LY294002 or luteolin could block CoCl_2_‐mediated expression of p‐Akt, HIF‐1α, and EMT typical marker E‐cadherin in A375 and B16‐F10 cells. Additionally, A375 and B16‐F10 cells cotreatment with luteolin and LY294002 could dramatically block CoCl_2_‐induced expression of p‐Akt, HIF‐1α, and E‐cadherin. These results suggest that Akt/HIF‐1α/EMT signaling pathway may play a crucial role in luteolin suppress metastasis.

## 4 DISCUSSION

Metastatic spread is still the leading cause of the melanoma death. Metastasis is a programmed multistep process, including cell migration, invasion, and adhesion. The exploration of effective natural compounds targeting EMT and angiogenesis have been the efficient therapeutic approach for the treatment of metastatic cancers (Yang et al., 2017). Luteolin, a flavonoid that is found in more than 300 plant species, has recently been shown to inhibit a variety of cancers, both in vitro and in vivo, with little to minimal toxicity (Lu et al., 2015; Ruan et al., 2012). Previous studies have shown that luteolin inhibits metastasis of triple‐negative breast cancer cells through suppressing EMT (Cook et al., 2017). In addition, luteolin inhibited the migration and invasion of HGF‐induced HepG2 cells by suppressing the phosphorylation of c‐Met, ERK, and Akt in hepatoma (Lee, Wu, Chen, Wang, & Tseng, 2006). However, a limited number of studies have examined luteolin's metastasis inhibitory effects of luteolin on melanoma. In this study, luteolin is prone to inhibit the metastasis of melanoma cells and tube formation of HUVECs. Concurrently, the underlying molecular mechanism of luteolin anti‐metastasis were investigated in association with inhibition of HIF‐1α/VEGF signaling pathway and the resulting decreased EMT and angiogenesis.

First, we performed MTT assay to evaluate the inhibitory effects of luteolin on the proliferation of A375 and B16‐F10 cells. The results demonstrated that luteolin significantly inhibited the proliferation of A375 and B16‐F10 cells in a time‐dependent and dose‐dependent manner. In order to eliminate the impact of luteolin's cytotoxic effect on cells metastatic ability, we chose the no apparently cytotoxic concentration 5, 10, and 20 μM of luteolin for further investigation. The metastasis potential of cancer cells is mainly determined by the ability of migration, invasion, and adhesion, which is commonly tested utilizing wounding healing, transwell systems with matrigel‐coated filters and adhesion assay. Thus, wound‐healing assay, transwell invasion assay, and adhesion assay were performed to examine the anti‐metastasis ability of luteolin. These results demonstrated that luteolin effectively attenuated the migration, invasion, and adhesion abilities of A375 and B16‐F10 cells. Meanwhile, the capillary tube formation of HUVECs on matrigel was performed to investigate the effect of luteolin on the process of angiogenesis in vitro. It was observed that luteolin significantly inhibited capillary tube formation of HUVECs.

Recent studies have validated the association between the metastasis potential of cancer cells and the activation of the EMT (Yi et al., 2018). Actually, the suppression of EMT is emerging as a common mechanism underlying the inhibitory effect on metastasis potential of cancer cells (Ryu et al., 2017). The first step of metastasis is cancer cells detach from the original tumor sites. E‐cadherin plays the role of the regulator of adhesions between cells. The decreased expression of E‐cadherin renders the cancer cells detached the original tumor sites more easily. Low E‐cadherin expression is positively associated with increased metastasis of cancer cells and poor prognosis. On the contrary, the increased expression of N‐cadherin and vimentin are associated with increased metastasis potential of multiple epithelial cancer cells. In this study, luteolin was found to significantly reduce the EMT process in both A375 and B16‐F10 cells, which suggest that the decreased expression of E‐cadherin and the increased expression of N‐cadherin and vimentin were alleviated by luteolin.

Tumor angiogenesis is another physiological process essentially required for tumor progression and metastasis (Shi, Chen, Wang, Guo, & Wang, 2018). Although increasing evidence indicates that angiogenesis is a highly sophisticated and coordinated process, the activation of a hypoxic‐induced factor (HIF) growth factor pathway remains the key modulator (Martinez‐Garcia et al., 2017). Tumor tissue is usually accompanied by hypoxia, which promotes HIF production. HIF‐1 is a dimeric transcription factor composed of the HIF‐1α and HIF‐1β subunits. HIF‐1α is rapidly degraded under normoxic conditions and stabilized under hypoxia, whereas HIF‐1β is expressed constitutively. HIF‐1α has been correlated with tumor grade, metastasis, and poor prognostic outcomes in various cancers (Mouriaux et al., 2014). In addition, HIF‐1α was shown to induce EMT in many types of cancer tissues. HIF‐1α expression profile was correlated with the expression levels of E‐cadherin, N‐cadherin, and vimentin. VEGF binds to its receptor activating VEGFR2, which further leads to the secretion of MMPs and the activation of Akt pathway, resulting in the metastasis. The degradation of ECM by MMPs mainly MMP‐2 and MMP‐9 have been consistently correlated with migration, invasion, and adhesion as well as angiogenesis in many types of cancer including melanoma (Akhavan, Karimi, Ghodrati, & Falahtpishe, 2011). Based on our observations in present study, luteolin may play a direct role in the degradation of HIF‐1α and subsequent VEGF signaling in melanoma cells, leading to the inhibition of hypoxia‐induced tumor angiogenesis. To the best of our knowledge, this is the first time report showing the anti‐metastatic and anti‐angiogenic potential of luteolin in melanoma at lower concentrations, without any noticeable cytotoxicity.

In summary, this study is of significant importance as currently limited therapeutic options are available for highly metastatic melanoma patients. Importantly, we found that luteolin inhibited melanoma cell migration, invasion, adhesion, and capillary tube formation abilities by inhibiting the HIF‐1α/VEGF signaling pathway. We propose that luteolin possesses a strong therapeutic potential against highly metastatic melanoma cells and could be an effective supplement for patients, and more detailed investigation regarding its mechanism of action and clinical trials involving its use are warranted.

## CONFLICT OF INTEREST

The authors declare that they have no conflicts of interest.
